# Suppressing autophagy: a strategy by *Escherichia coli* O157:H7 for its survival on host epithelial cells

**DOI:** 10.1038/s41419-017-0095-3

**Published:** 2018-01-19

**Authors:** Yansong Xue, Mei-Jun Zhu

**Affiliations:** School of Food Science, Washington State University, Pullman, WA 99164 USA

## Introduction

The primary function of autophagy is to degrade aged or impaired organelles, thus allowing cells to survive under extreme environments and rejuvenate cellular function. Besides the self-fulfillment function, autophagy also serves as a defense mechanism to invasive bacteria. Upon infection, autophagy is activated to engulf vesicles containing pathogenic bacteria for degradation. Conversely, to persist in host cells, coevolved pathogens develop sophisticated tactics to evade the trap of autophagy using various strategies. A recent publication shows that RavZ, a cysteine protease-like factor of intracellular pathogen *Legionella pneumophila* decreases the microtubule-associated protein 1 light chain-3B II (LC3BII) level by irreversibly blocking the conversion from LC3BI to LC3BII^1^. Likewise, pathogenicity islands 2 (SPI-2) of *Salmonella* Typhimurium manipulates host autophagy by actively regulating focal adhesion kinase (FAK)^2^.

*Escherichia coli* O157:H7 is a gram-negative extracellular pathogen that produces Shiga toxins causing hemorrhagic diarrhea and hemolytic-uremic syndrome. In addition, it contains type 3 secretion system (T3SS) apparatus and effectors such as translocated intimin receptor (Tir) and *E. coli* secreted proteins (Esps). These effectors lead to intimate adhesion to host intestinal epithelial cells and formation of attachment and effacement (A/E) lesions^3^.

**Fig. 1:**
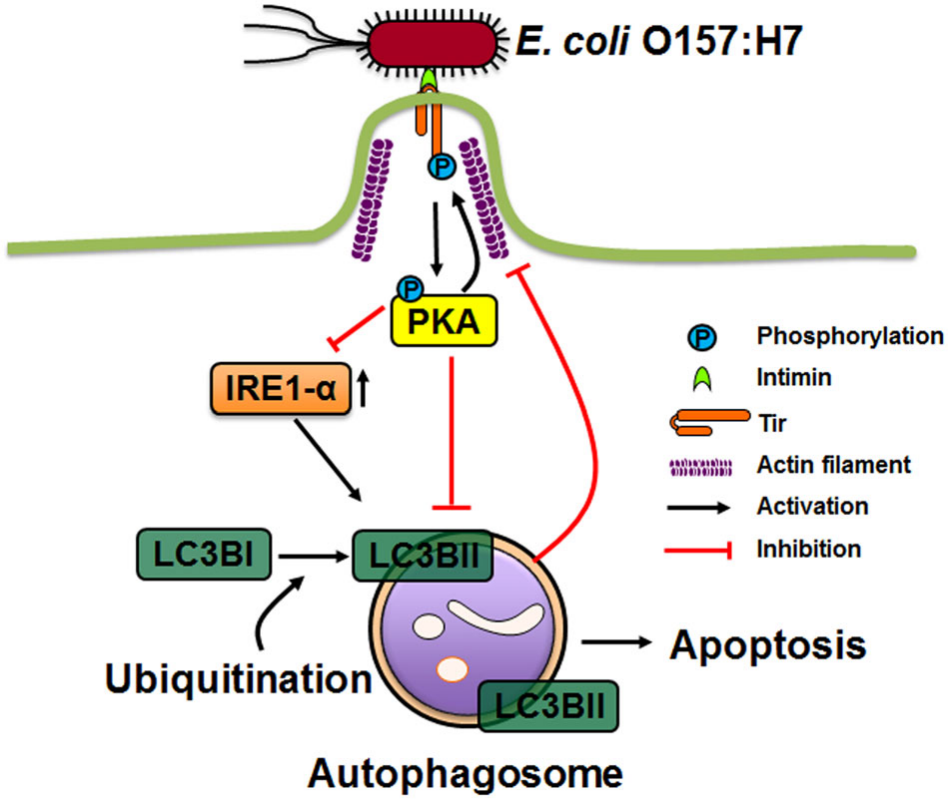
Schematic diagram of autophagy inhibition by *E. coli* O157:H7 Adhesion of *E. coli* O157:H7 activates PKA through translocated intimin receptor (Tir), which inhibits endoplasmic reticulum stress, and blocks autophagosome formation. Activation of autophagy disturbs the adhesion of *E. coli* O157:H7 to host cells with an undetermined mechanism.

Despite evidence that manipulation of the host autophagic process is critical in the pathogenesis of invasive pathogens, the interaction of autophagy with extracellular pathogen *E. coli* O157:H7 is sparsely studied. Recent publications show that Shiga toxin serves as an autophagy inducer in immune cells and intestinal epithelial cells, likely through the induction of endoplasmic reticulum (ER) stress^4,5^; Shiga toxin 2 induces DNA-damage-inducible transcript 3 (DDIT3) activation and Akt1 inactivation, further leading to autophagic cell death^4^. Our recent publication in *Cell Death Discovery* reported that *E. coli* O157:H7 subverted host autophagic response to promote epithelial adhesion, which was mediated by T3SS effector, Tir^6^ (Fig. 1). *E. coli* O157:H7 infection inhibited autophagy activity in colonic epithelial cells, which was abolished by *tir* deletion and was further restored by complementary *tir* plasmid expression^6^.

Tir has multiple residues that can be modified by host protein kinases. Upon adhesion to the host cells, Tir recruits various host protein kinases that facilitate epithelial adhesion, potentially interfering with host autophagy. Besides tyrosine phosphorylation, Tir is subjected to serine phosphorylation by host kinases such as cAMP-activated protein kinase A (PKA)^7^. We observed a robust PKA activation in response to *E. coli* O157:H7 infection but not in *tir* deletion mutant (Δ*tir*) strain infected cells, indicating Tir mediated PKA activation. We further showed that PKA activation was essential for *E. coli* O157:H7-induced autophagy inhibition, blocking PKA abolished *E. coli* O157:H7 ability to inhibit autophagy^6^. Correspondingly, cholera toxin and edema toxin from *Vibrio cholerae* and *Bacillus anthracis*, respectively, induce PKA hyperactivation and reduce autophagosome formation^8^. The activation of PKA specifically phosphorylates autophagic proteins Atg1 and Atg13 and impairs their localization to phagophore^9^. *S*. Typhimurium selectively activates non-receptor tyrosine kinase FAK, thereby retarding the recruitment of LC3B to the autophagosomes^2^. The above data show that host protein kinases are hijacked by bacterial factors and play mediatory roles in autophagy regulation.

*E. coli* O157:H7 infection causes the disturbance of ER function^4^. ER stress is a potent inducer in autophagy activation resulting from accumulation of unfolded protein response (UPR)^10^. The ER stress sensor inositol-requiring enzyme 1α (IRE1α) is one of the mediators of UPR and has been implicated in ER stress-induced autophagy^11^. In our recent publication, upon *E. coli* O157:H7 infection, IRE1-α was decreased^6^, which may reduce autophagy-induced cell death and benefit bacterial survival. Accumulating studies have indicated that autophagy-induced cell death is a host strategy to inhibit bacterial replication. Macrophage apoptosis caused by *Salmonella* infection is highly associated with autophagy hyperactivation induced by T3SS factor SipB^12^, which halts the replication of intracellular *Salmonella* effectively. Corresponding to inhibited autophagy, *E. coli* O157:H7 infection inhibited caspase-8 and poly (ADP-ribose) polymerase (PARP) cleavage, suggesting that infection decelerated cell death process^6^.

Our recent publication in *Cell Death Discovery* further showed that host autophagy activation by starvation or rapamycin reduced the adherence of *E. coli* O157:H7 to HT-29 cells, while autophagy inhibition by chloroquine enhanced its epithelial adhesion^6^. It is consistent with our previous publication that tumor necrosis factor (TNF) reduced *E. coli* O157:H7 attachment to epithelial cells, which may be partially due to the activated autophagy^13^. Collectively, these data suggest that inhibition of host autophagy favors bacterial interaction with host cells; however, the detailed mechanism of how autophagy affects bacterial attachment warrants further research.

In summary, our recent paper in *Cell Death Discovery* demonstrates that *E. coli* O157:H7 has evolved strategies to block host autophagic response partially via activation of PKA that promotes its epithelial adhesion. Our findings provide new insight in the fight against *E. coli* O157:H7 infection. The drugs/chemicals targeting PKA activation or boosting autophagy activity could be used to reduce/eliminate *E. coli* O157:H7 gut colonization and infection.
